# At the centre of neuronal, synaptic and axonal pathology in murine prion disease: degeneration of neuroanatomically linked thalamic and brainstem nuclei

**DOI:** 10.1111/nan.12232

**Published:** 2015-05-30

**Authors:** Renata Reis, Edel Hennessy, Caoimhe Murray, Éadaoin W. Griffin, Colm Cunningham

**Affiliations:** ^1^ Trinity College Institute of Neuroscience and School of Biochemistry and ImmunologyTrinity College DublinDublinIreland

**Keywords:** axon, cathepsin D, chronic neurodegeneration, neuroanatomical spread, phagocytosis, synapse

## Abstract

**Aims:**

The processes by which neurons degenerate in chronic neurodegenerative diseases remain unclear. Synaptic loss and axonal pathology frequently precede neuronal loss and protein aggregation demonstrably spreads along neuroanatomical pathways in many neurodegenerative diseases. The spread of neuronal pathology is less studied.

**Methods:**

We previously demonstrated severe neurodegeneration in the posterior thalamus of multiple prion disease strains. Here we used the ME7 model of prion disease to examine the nature of this degeneration in the posterior thalamus and the major brainstem projections into this region.

**Results:**

We objectively quantified neurological decline between 16 and 18 weeks post‐inoculation and observed thalamic subregion‐selective neuronal, synaptic and axonal pathology while demonstrating relatively uniform protease‐resistant prion protein (PrP) aggregation and microgliosis across the posterior thalamus. Novel amyloid precursor protein (APP) pathology was particularly prominent in the thalamic posterior (PO) and ventroposterior lateral (VPL) nuclei. The brainstem nuclei forming the major projections to these thalamic nuclei were examined. Massive neuronal loss in the PO was not matched by significant neuronal loss in the interpolaris (Sp5I), while massive synaptic loss in the ventral posteromedial nucleus (VPM) did correspond with significant neuronal loss in the principal trigeminal nucleus. Likewise, significant VPL synaptic loss was matched by significant neuronal loss in the gracile and cuneate nuclei.

**Conclusion:**

These findings demonstrate significant spread of neuronal pathology from the thalamus to the brainstem in prion disease. The divergent neuropathological features in adjacent neuronal populations demonstrates that there are discrete pathways to neurodegeneration in different neuronal populations.

## Introduction

Understanding chronic neurodegenerative disease is one of the most significant challenges we face as our population ages in the coming decades. It is now widely recognized that loss of presynaptic terminals is a key neurodegenerative event that precedes loss of the neuronal cell soma in many neurodegenerative diseases [1] and axonal dysfunction is also known to accompany or precede cell loss in many of these states. Diseases such as Alzheimer's disease, Huntington's disease, Parkinson's disease and prion diseases are known to spread along neuroanatomical pathways, in a prion‐like manner [2, 3], but the relationship between pathology in different neuroanatomical regions has usually been examined at the level of deposition of intracellular (tau, α‐synuclein) or extracellular (Aβ and PrP^Sc^) proteins [4, 5, 6]. It is unclear whether loss of synapses in one region necessarily leads to neuronal loss in the area from which those terminals arise and whether neuronal pathology in one region has predictable effects on projecting and target cells.

Prion diseases are a group of transmissible spongiform encephalopathies, fatal neurodegenerative diseases of mammals characterized by the deposition of protease‐resistant amyloid (PrP^Sc^) on neurite membranes and in the extracellular space [7], robust synaptic loss or dendritic loss and neurodegeneration along neuroanatomical pathways [3, 6, 8, 9]. However, the mechanisms and regional specificity of neurodegeneration remain unclear and most neuropathological profiling in rodent models is assessed by PrP^Sc^ deposition and vacuolation scoring, providing a semi‐quantitative description of spongiform change in nine regions of the brain [10]. Studies of loss/degeneration of specific neuronal populations has been relatively limited, with the hippocampus and cerebellum being frequently quantified [3, 8, 11, 12]. There is evidence for different mechanisms of degeneration (presynaptic terminal *vs.* post‐synaptic dendrite) occurring in neurons of different brain regions [12], but the neuroanatomical spread of neuronal pathology remains little studied.

The ME7 model of prion disease offers a tractable model to study neuroanatomical spread of neuronal pathology. We have identified very significant thalamic pathology in mice inoculated with ME7, 79A or 22L with robust neuronal loss in the posterior areas of the thalamus at later stages of disease in ME7, 79A and 22L strains [13] and PrP^Sc^ deposition, astrocytosis and microgliosis occurring at early stages (12 weeks) [14]. There is evidence for pathology in the thalamus in prion disease in other experimental animals [15, 16, 17] and in the multiple forms of human prion disease including new variant and sporadic Creutzfeldt–Jakob diseases (sCJD) [18, 19], fatal familial insomnia (FFI) [20, 21] and Gerstmann‐Straussler‐Scheinker (GSS) disease [22]. The thalamus is of particular importance in FFI, an autosomal dominant prion disease characterized by progressive insomnia and prominent autonomic alterations, and both thalamic hypometabolism and neuronal death are key features of the disease [21] occurring more than 1 year before the clinical presentation [20]. Thus, the thalamus shows significant pathology in both rodent and human prion diseases. The thalamus is a subcortical structure that is very widely connected within the brain and the very significant pathology observed in thalamic regions predicts that the major connections to and from the thalamus will also show significant degeneration in parallel with thalamic degeneration.

We have previously examined neuropathology in multiple brain regions, in multiple strains at key time points in the progression of prion disease [8, 13, 14]. Here, our aim is to characterize thalamic pathology in ME7 mice when significant neurodegeneration has occurred in this structure, which coincides with failure on motor coordination and muscle strength tasks, demonstrating that these mice are beginning the terminal stages of disease (18 weeks post‐inoculation). Specifically, we investigated thalamic synaptic, neuronal, microglial, APP and PrP pathology in the posterior thalamic nuclei and then went on to examine major brainstem regions connected with these specific thalamic structures for similar features of pathology.

## Materials and methods

### Animals and stereotaxic surgery

Female C57BL/6 mice (Harlan Olac, Blackthorn, UK) were kept in groups of five in plastic cages in a temperature‐controlled room (21°C) with a 12:12 h light‐dark cycle (light on from 08:00 to 20:00 h). They had free access to food and water. They were anaesthetized with an intraperitoneal injection of Avertin (2,2,2‐tribromoethanol 50% w/v in tertiary amyl alcohol, diluted 1:40 in H_2_O; 20 ml/kg, i.p.) and stereotactically injected bilaterally with 1 μl of a 10% w/v scrapie (ME7 strain)‐infected C57BL/6 brain homogenate at co‐ordinates from bregma (anteroposterior − 2.0 mm; mediolateral − 1.6 mm; dorsoventral – 1.7 mm) using a Hamilton microsyringe. Control animals were injected with a 10% w/v normal brain homogenate (NBH) in normal saline, derived from a normal C57BL/6 mouse. We assessed motor coordination and muscle strength weekly, for 60 s on bar and screen apparatus, as previously described [13]. All animal procedures were done under licence from the Irish Department of Health after a full ethical review by the TCD animal research ethics committee and performed in compliance with the Cruelty to Animals Act, 1876 and the European Community Directive, 86/609/EEC.

### Tissue processing, immunohistochemistry and digital analysis

Animals were terminally anaesthetized with sodium pentobarbital and transcardially perfused with heparinized saline followed by 10% formal saline. Brains were paraffin embedded and 10 μm coronal sections through the dorsal hippocampus, thalamus, principal trigeminal nucleus (PrTN), interpolar part of spinal trigeminal nucleus (Sp5I) and the gracile and cuneate nuclei were cut on a microtome, dewaxed in xylene and rehydrated. Immunohistochemistry was performed for β‐amyloid precursor protein (APP, Life Technologies, MS, USA), synaptophysin (SY38) and neuron‐specific nuclear protein (NeuN, Millipore, Carrigtwohill, Ireland), ubiquitin (DAKO, Dublin, Ireland), hyperphosphorylated tau (AT8, Thermo Scientific, MS, USA), cathepsin D (Santa Cruz Biotechnology, CA, USA), IBA‐1 (Abcam, Cambridge, UK), PrP (6D11, Santa Cruz Biotechnology) and both phosphorylated (SMI‐31, Abcam) and nonphosphorylated (SMI32, Covance, NJ, USA) neurofilament H. Biotinylated secondary antibodies, normal sera, mouse‐on‐mouse blocking kit and avidin‐biotin complex were purchased from Vector Laboratories (Peterborough, UK). Immunohistochemistry was carried out by the avidin‐biotin complex (ABC) method, with 0.015% v/v hydrogen peroxide as the substrate and visualized with diaminobenzidine (DAB). Primary antibody specific modifications are detailed below. Coverslipped slides were digitally captured with an Olympus DP25 camera (Mason, Dublin, Ireland) mounted on a Leica DM3000 microscope (Laboratory Instruments and Supplies, Dublin, Ireland), captured using Cell A^™^ software (Olympus, Mason, Dublin, Ireland) and analysed using ImageJ software (NIH; http://imagej.nih.gov/ij/).

#### NeuN

Rehydrated sections were treated with 1% H_2_O_2_ to block endogenous peroxidase activity, followed by citrate treatment, involving microwaving at full power in 10 mM citrate buffer, pH 6, for 5 min followed by 5 min cooling and a further 5 min microwaving. Sections were washed with phosphate buffered saline (PBS) solution and were blocked with 10% normal horse serum and incubated overnight with anti‐NeuN (1:5000) at 4°C. Sections were then incubated with biotinylated horse antimouse (1:200) for 45 min and the antigen was visualized and the sections mounted as above. Immunohistochemically labelled identical sections of PrTN were photographed at 40×. Subregions of PrTN: four sections of PrTNvl and PrTNdm from six to eight animals per group were analysed. Samples were photographed on the same light and colour settings and the images were converted to 8‐bit, thresholded to create binary images, optimizing the capture of neurons. Number of neurons and total area (%) were calculated using ImageJ software using the ‘analyse particles’ function applied to each picture of NBH and ME7 animals considering the parameters previously defined (size: 250–infinity and circularity: 0–1). For the thalamic neuronal quantification, serial sections were captured at 20× magnification (area of 0.29 mm^2^). The number of neurons was defined by manual counting of two sections from each animal. Quantification of gracile and cuneate nuclei were performed by counting of all NeuN‐positive cells in each region in 3–4 sections of four animals for each group.

#### PrP

Sections were stained to demonstrate the presence of the abnormal prion protein in the ME7 mouse model. Following de‐waxing and rehydration, sections were processed using hydrated autoclaving at 121°C for 20 min and cooled to room temperature by the slow addition of cold tap water. Sections were treated with 90% formic acid for 5 min and quenched for 20 min. Sections were incubated in Proteinase‐K for 30 min to aid degradation of nonscrapie form cellular PrP. Sections were blocked with 10% NHS before overnight incubation at 4°C with the mouse antibody 6D11 (1/100). Sections were then incubated in biotinylated horse antimouse secondary (1/250) and developed using the ABC method and DAB as described above.

#### IBA‐1

Rehydrated identical brainstem sections were treated with 1% H_2_O_2_ to block endogenous peroxidase activity and pretreated by the citrate method as before. Sections were washed with PBS solution and were treated with pepsin 0.04% diluted in HCl 0.1 M for 15 min. Sections were then washed in PBS, blocked with 10% normal rabbit serum and incubated overnight with anti‐IBA‐1, (Abcam, 1:2000 at 4°C. Sections were incubated with biotinylated rabbit antigoat (1:100) for 45 min and the antigen was visualized as described above.

#### APP

Rehydrated sections were treated with 1% H_2_O_2_ to block endogenous peroxidase activity, followed by citrate treatment as described above. Sections were washed with the addition of 0.1% Tween to the PBS solution, were blocked with 10% normal horse serum and incubated overnight with anti‐APP (Invitrogen 13–0200, 1:100) at 4°C. Sections were then incubated with biotinylated horse antimouse (1:200) for 45 min. The antigen was visualized as above. Following this, sections were then counterstained in Harris' haematoxylin (VWR, Dublin, Ireland) and dehydrated before coverslipping with DPX.

Immunohistochemically labelled sections were captured at 40×. Four sections of PrTN from 3–4 animals from each group were analysed as follows: pictures were processed to remove the Harris' haematoxylin counterstaining using the separate blue on image tool of Cell A software and saved as a new picture; the resulting pictures were opened in ImageJ and thresholded such that only APP‐positive labelled spheroids had sufficient transmittance to be retained. Spheroids were then assessed using the ‘analyse particles’ function using predefined parameters (size: 5–500 and circularity: 0.7–1). The mask tool allowed depiction of the selected particles for analysis. All samples were photographed on the same light and colour settings to minimize any influences of microscopy and camera settings.

#### Synaptophysin

Rehydrated sections were treated for 30 min with 0.2 M boric acid, pH 9, 65°C and incubated with 1% H_2_O_2_/PBS for 15 min to eliminate nonspecific peroxidase activity. Sections were washed in PBS and blocked with 10% normal horse serum. Sections were then incubated overnight with antisynaptophysin (Millipore, 1:2000) at room temperature. Thereafter, sections were washed with PBS and incubated with biotinylated horse antimouse secondary antibody (1:200). The DAB reaction was carried out with the addition of ammonium nickel sulphate (0.04% w/v) to enhance the intensity. The sections were dehydrated and mounted in DPX. The density of synaptophysin staining was quantified by pixel density analysis on digitally captured images using ImageJ image analysis software (NIH, USA, http://imagej.nih.gov/ij/) using a similar method to that which we previously published [8]. In the PrTN, ventrolateral part (PrTNvl), the average of three small areas of uniform Sy38 labelling was calculated, whereas a single larger area was selected in the PrTN, dorsomedial part (PrTNdm). These were quantified by pixel density analysis. The spinal trigeminal tract (the white matter of highest transmittance) was used as internal standard and all other transmittances were subtracted from this. Four sections per animal from five animals per group were assayed. The transmittance for each region was defined as a contrast ratio as follows:

$$\begin{array}{l} \text{Ratio}\;\text{for}\;\text{PrTNvl}\\ \;=\left(\text{white}\;\text{matter}-\text{PrTNvl}\right)/\left(\text{white}\;\text{matter}+\text{PrTNvl}\right) \end{array}$$

$$\begin{array}{l} \text{Ratio}\;\text{for}\;\text{PrTNdm}\\ \;=\left(\text{white}\;\text{matter}-\text{PrTNdm}\right)/\left(\text{white}\;\text{matter}+\text{PrTNdm}\right) \end{array}$$

#### SMI‐31

Sections were labelled for 200 kDa phosphorylated neurofilament using SMI‐31 (Abcam; ab24570, 1:1000). Sections were quenched for 20 min in methanol 1% H_2_O_2_ to block endogenous peroxidase activity followed by citrate pretreatment as described above. Sections were blocked using 10% NHS and incubated with primary antibody overnight at 4°C before continuing with appropriate secondary antibody, ABC and DAB reaction as above.

#### SMI32

Rehydrated identical brainstem sections were treated with 1% H_2_O_2_ to block endogenous peroxidase activity followed by citrate pretreatment as above. Sections were washed with the PBS solution and were blocked with 10% normal horse serum and incubated overnight with anti‐SMI32 (Covance, 1:500) at 4°C. Sections were incubated with biotinylated horse antimouse (1:100) for 45 min. The antigen was visualized and the sections mounted as above.

### Confocal microscopy

Double‐labelling experiments were performed using primary antibodies against APP (Invitrogen 13–0200), cathepsin D (Santa Cruz SC‐6486), SMI‐31 (Abcam ab24570) and IBA‐1 (Abcam ab5076). Alexa Fluor secondary reagents (Invitrogen Biosciences) included donkey antigoat (488), donkey antimouse (568) and a Zenon™ mouse IgG labelling kit (488: Z25060, Thermo Scientific, MS, USA). For APP double‐labelling with cathepsin D or IBA‐1, antigen retrieval was performed by microwaving in citrate as before. Sections were washed and treated for 20 min with 0.04% pepsin in 0.1 M HCl. Sections were washed and then blocked in 10% normal donkey serum. Primary antibodies for cathepsin D (1/500) and IBA‐1 (1/2000) were incubated overnight. The following day, sections were incubated in 488 donkey antigoat secondary and incubated overnight with anti‐APP primary antibody (1/100). The following day 568‐donkey antimouse secondary was added, sections were treated with Hoechst 33258 nucleic acid stain (Invitrogen Biosciences, excitation 352 nm, emission 461 nm), mounted in ProLong Gold (Invitrogen Biosciences) and coverslipped. For APP co‐localization with SMI‐31, antigen retrieval was performed using the citrate method. Sections were washed and then blocked in 10% normal donkey serum. Primary antibody for SMI‐31 (1/1000) was incubated overnight. The following day, sections were incubated with 568 donkey antimouse IgG and treated overnight with the Zenon‐labelled primary antibody against APP (1/100). This was followed by 3× 10‐min PBS‐Tween washes, 1× 5‐min PBS, 15 min PFA fixation and 2× 15‐min PBS wash. Sections were treated with Hoechst 33258 nucleic acid stain (Invitrogen), mounted in ProLong Gold (Invitrogen Biosciences) and cover‐slipped. Double‐labelled sections were visualized and captured on an Olympus FV1000 confocal microscope with a 60× oil objective (numerical aperture, 1.35) using sequential excitation. Emission filters were applied at 425–450 nm for Hoechst 33258, at 500–530 nm for Alexa fluor 488‐ and Zenon‐labelled antibodies and at 560–700 nm for Alexa Fluor 568‐labelled antibodies.

### Statistics

Graphs were prepared and data were analysed using GraphPad Prism 5 software (GraphPad Software Inc., CA, USA). The data (ME7 *vs.* NBH) were compared by Student's *t*‐test and values of *P* < 0.05 were considered statistically significant. All data are shown as mean ± SEM.

## Results

### Neurological decline and neuronal loss

**Figure 1:**
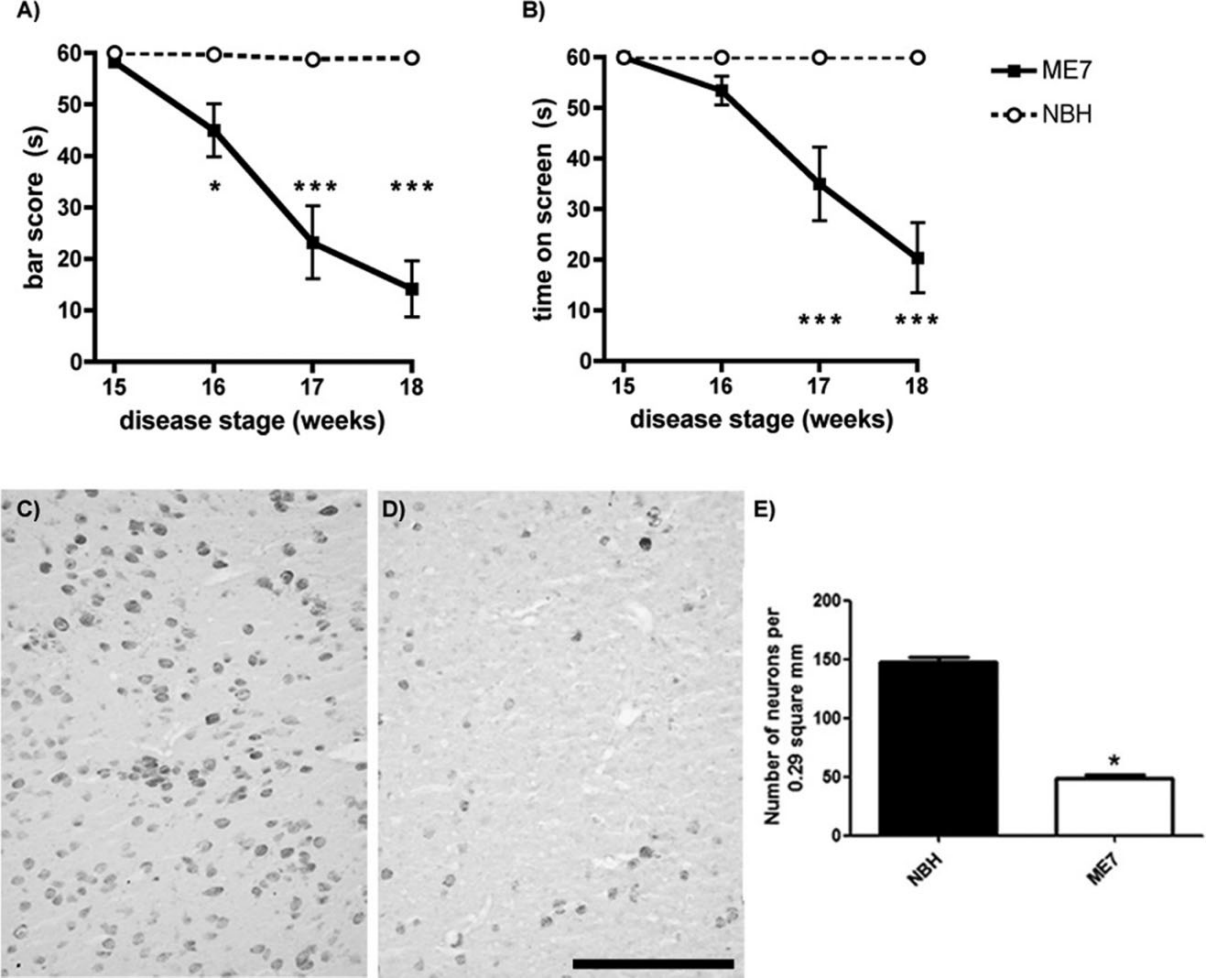
Neurological decline and neuronal loss. Longitudinal measures of performance on the horizontal bar (A) and inverted screen (B) tasks. *n* = 10 in each group, *denotes *P* < 0.05, ***P < 0.001 by Bonferroni post‐hoc after significant two‐way anova. (C,D) Immunostaining for NeuN in posterior thalamus of NBH and ME7 animals, respectively. Strong qualitative reduction of neurons was found in ME7 animals, which was confirmed by quantitative analysis shown on the graph (E). *t‐test, *P* < 0.001. Data are presented as mean ± SEM, *n* = 10 ME7 and 6 NBH. Scale bar: 200 μm.

We monitored the animals weekly on horizontal bar and inverted screen tests of motor coordination and muscle strength (Figure 1 **A,B**). ME7 animals showed a clear progressive decline from an intact baseline at 15 weeks to very significant impairment at 18 weeks (interaction between disease and time by two‐way anova *F* ≥ 18.6, df = 3.54, *P* < 0.0001) Deficits were significant from 16 weeks on the bar (*P* < 0.05; Bonferroni post‐hoc) and from 17 weeks on the screen (*P* < 0.001).

To assess thalamic pathology at the 18‐week stage of ME7 disease progression, posterior thalamus (PO) pictures (area of 0.29 mm^2^) were obtained from NBH and ME7 experimental groups were immunolabelled for NeuN and quantified (Figure 1 **C–E**). Very marked neuronal loss (>60%) is evident in ME7 animals compared with the relatively uniform neuronal population present in NBH animals (*P* < 0.001). This confirms the robust thalamic neuronal death, in particular in the posterior nucleus, previously reported in this model.

### Thalamic pathology

**Figure 2:**
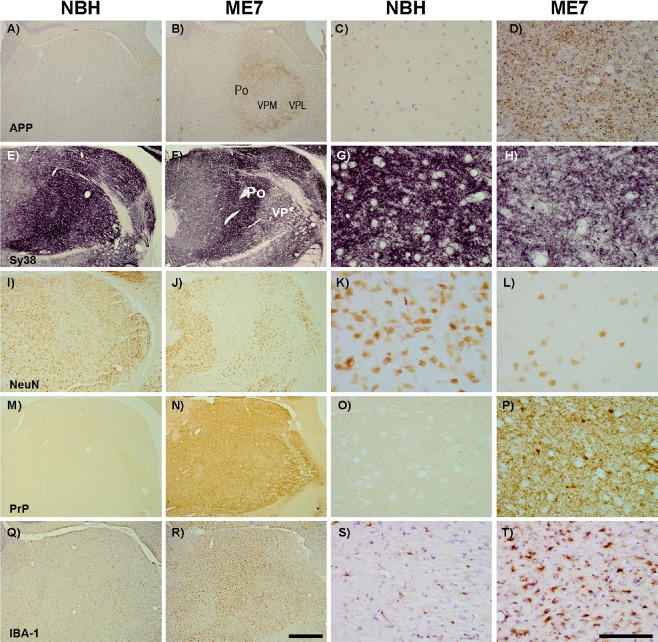
Subregion selective thalamic pathology. Photomicrographs of thalamic regions (Po, VPM and VPL) immunostained for APP, Sy38, NeuN, PrP and IBA‐1 of NBH (A,E,I,M,Q) and ME7 (B,F,J,N,R) animals with respective photomicrographs on high magnification (NBH: C,G,K,O,S); ME7: D,H,L,P,T). Strong labelling was observed for APP (A *vs*. B) in the posterior thalamus (Po) and the VPL, but less so in the VPM. Sy38 showed robust synaptic loss but this was less obvious in the Po than in the VP areas (E *vs*. F). Strong loss of NeuN‐positive neurons was observed in PO but less obviously in VP areas (I *vs*. J). Conversely, PrP (N) and IBA‐1‐positive microglial activation (R) did not show any apparent subregion specificity. Scale bars of low and high power: 500 μm and 100 μm, respectively.

**Figure 3:**
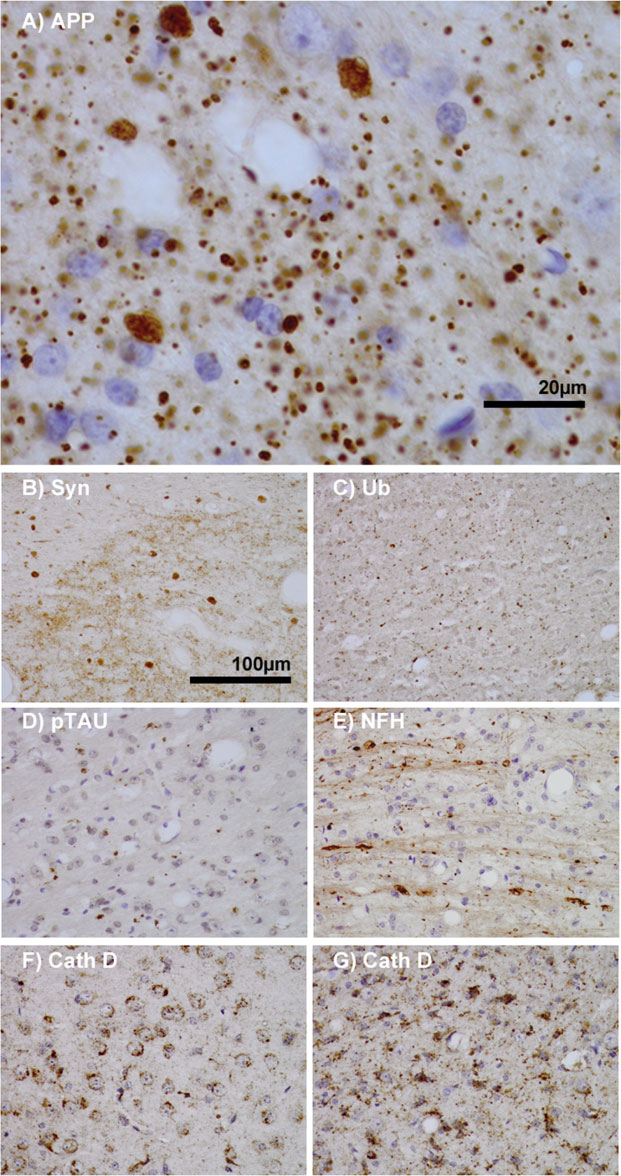
Features of thalamic pathology. (A) High power (100× objective) photo‐micrographs of thalamic APP pathology shows large numbers of 1–2 μm diameter positive elements as well as smaller numbers of much larger spheroids (>6 μm). For comparative purposes, a number of other pathological features were examined for similarities and these were photographed at 40×. (B) Sy38 shows large spheroids of similar size to APP and significant overall loss, but no small deposits. (C) Ubiquitin shows large numbers of small ‘dot‐like’ deposits that are similar in size but very much fewer that those observed with APP. (D) Phospho tau (AT8) labelling shows frequent apparently neuritic labelling. (E) SMI‐32, neurofilament H labelling showed strong spheroid labelling in axons emerging from the medial lemniscus but much fewer spheroids in the grey matter. (F) Cathepsin D showed typical lysosomal labelling of small spherical structures in a perinuclear location, predominantly visible in neurons of the PO in NBH animals. (G) Cathepsin D in ME7 animals showed predominantly glial localization and consisted of larger structures or accumulations in perinuclear but also in some glial processes.

To assess subregion‐specific neuropathological changes in infected animals, immunohistochemistry for five different markers was performed for NBH and ME7 animals as shown in Figure 2. A very marked feature of this pathology is a very intense labelling for the APP in the PO and ventral posterior lateral thalamus (VPL) in ME7 animals (Figure 2 **B,D**). These APP‐positive elements do not resemble axonal end‐bulbs or amyloid plaques. A minority of these are large axonal spheroids (approximately 4–8 μm, see also Figure 3 **A**) but the majority are small (1–3 μm), densely labelled and remarkably numerous puncta. They are also present, but considerably less numerous in the ventral posteromedial nucleus (VPM) in infected animals and there are no such deposits in the NBH group (Figure 2 **A,C**).

Synaptic density was, in general, compromised in ME7 group with significant loss of density in all three thalamic areas examined here (2 E *vs.* F). However, this synaptic loss is most severe in the VPL/VPM while the PO shows relative sparing, demonstrating a clear, but incomplete, dissociation between APP deposits and synaptic loss. Many Sy38‐positive spheroids are also evident in the ME7 animals, particularly in area of most extreme synaptic loss (VPM/VPL). These spheroids are very similar in form and size to some APP deposits although they are bigger and significantly less numerous than the majority of APP‐positive deposits (Figure 2 **D *vs.* H**).

Neuronal loss, examined by NeuN labelling, showed that the significant neuronal loss found in the PO nucleus of ME7 animals (Figure 2 **I *vs.* J and K *vs.* L**), is therefore strongly associated with the most numerous APP deposits (Figure 2 **B**), but not with the strongest synaptic loss [2]. That is, the PO is less compromised for synaptophysin immunolabelling than the VPM and VPL (Figure 2 **E,F**).

Conversely, immunohistochemistry for PrP^sc^ (Figure 2 **M–P**) showed the presence of PrP^Sc^ throughout all these subregions of the thalamus with no clear subregion or subcellular selectivity. Likewise, microglial activation was very robust across the posterior regions of the thalamus without obviously divergent patterns in the three subregions as illustrated using IBA‐1 immunolabelling (Figure 2 **Q–T**).

Collectively, these markers show that there are clear thalamic subregion selective neurodegenerative changes despite relatively uniform labelling with PrP^Sc^ and IBA‐1 in these regions.

### APP deposits

We performed further neuropathological labelling experiments in the thalamus to seek other markers showing a similar pattern of labelling to that observed with APP (Figure 3 **A**). Synaptophysin labelling is shown to emphasize the similarity between the larger spheroids labelled with APP and Sy38; however, this was most prominent in the VPL (Figure 3 **B**). Ubiquitin labelling demonstrated a wide distribution of small ‘dot‐like’ structures throughout the thalamus [3] that may correspond to the smaller end of the APP‐positive size spectrum, although clearly not as numerous as APP‐positive puncta. We also observed relatively frequent and widely distributed labelling of hyper‐phosphorylated tau using AT8 (Figure 3 **D**) and this appeared to be neuritic and did not strongly resemble the APP labelling. SMI32 labelling of dephosphorylated neurofilament heavy chain showed very clear axonal pathology and this was most clearly visible where axons emerge from the medial lemniscus into the PO (Figure 3 **E**), but once again, these did not resemble the majority of APP labelling. Finally, cathepsin D labelling demonstrated a clear shift from largely neuronal lysosomal labelling in NBH animals (Figure 3 **F**) to a predominantly glial lysosomal labelling in ME7 animals (Figure 3 **G**). Larger lysosomes appeared consistent in size with some of the smaller APP puncta.

**Figure 4:**
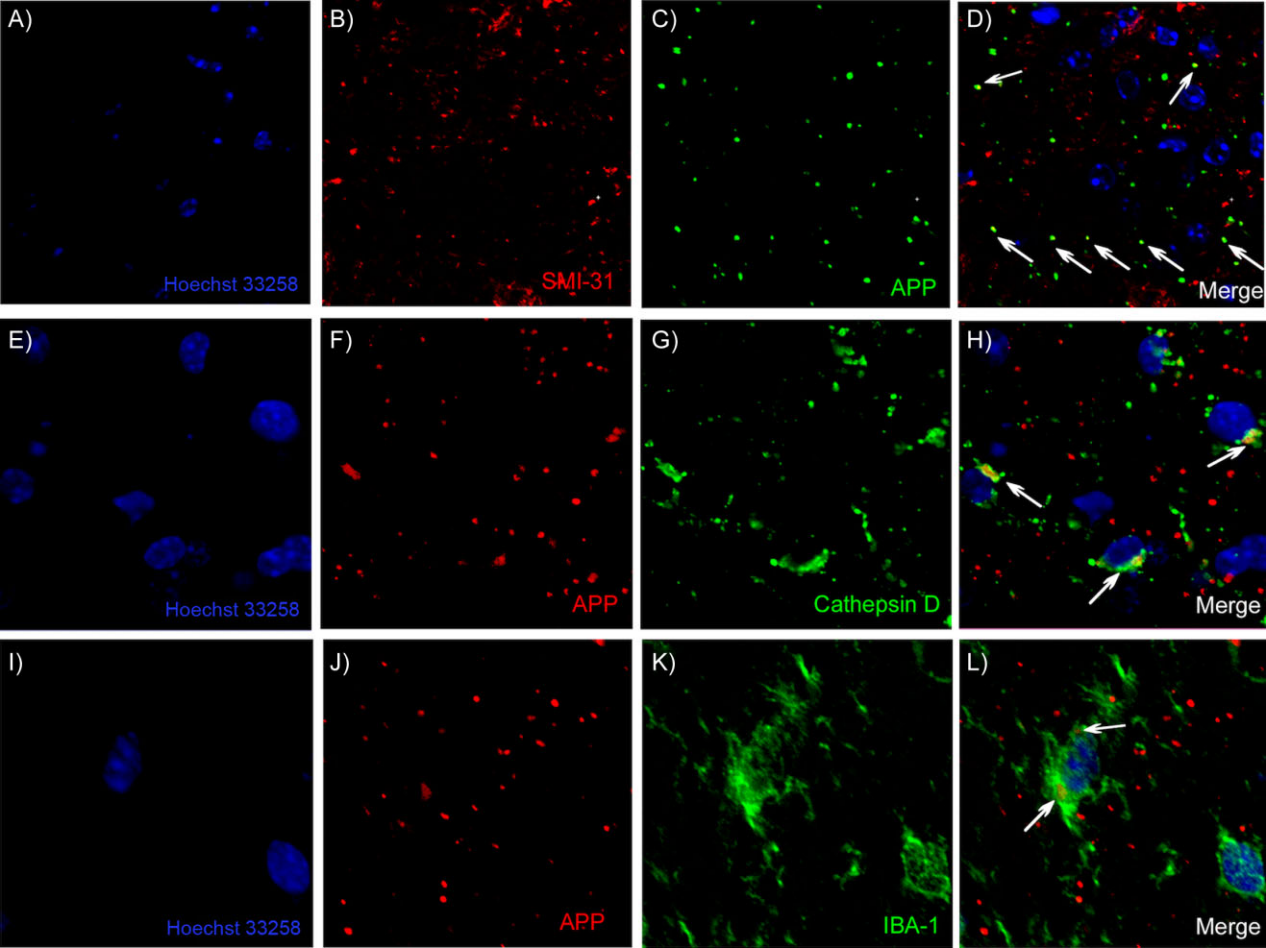
Identification of APP‐positive elements. Confocal microscopic images show the nuclear stain Hoechst 33258 (A, E and I) and APP‐positive elements as distributed puncta (C, F and J). Neurofilament heavy chain labelling with SMI‐31 (B) and APP‐positive elements (C) show some co‐localization in D. The lysosomal protease cathepsin D shows relatively condensed labelling in a perinuclear region of multiple cells (G) and a number of APP‐positive elements (F) co‐localize with cathepsin D (H). APP‐positive puncta (J) are also largely distinct from IBA‐1‐positive microglia (K) but some co‐localization with these microglia does occur (L). Areas of co‐localization are indicated with white arrows.

We performed double‐labelling immunohistochemistry with confocal microscopy to learn more about the observed APP deposits (Figure 4). In the area of maximum APP deposition and neuronal death, the PO, there were very few NeuN‐positive neurons remaining and we did not identify any co‐localization of APP with NeuN. However, many APP‐positive puncta were double labelled with the phosphorylated neurofilament heavy chain marker SMI‐31 and in some cases (Figure 4 **D**) these double‐positive puncta had a characteristic ‘beads on a string’ appearance suggestive of axonal spheroids within an intact axon (see also *Axonal injury* and Figure 8). Based on the hypothesis that in the absence of numerous neurons in the field with maximum puncta, APP puncta may be largely extracellular and prone to phagocytosis, we double labelled with antibodies against the lysosomal marker cathepsin D and identified many elements that were double‐labelled for APP and cathepsin D (Figure 4 **H**). This indicates presence of the APP within the lysosome, and according to the morphology of the associated nuclei, these cells appeared to include both microglia and astrocytes (Figure 4 **H**). The co‐localization of APP puncta with microglia was confirmed by double labelling with IBA‐1 (Figure 4 **L**). It is important to point out that the majority of APP‐positive puncta were not double‐labelled with SMI31, cathepsin D or IBA‐1 and may be extracellular. We were unable to perform co‐localization of APP with ubiquitin or synaptophysin due to incompatible antigen retrieval protocols.

### Projections to the thalamus: neuronal pathology

**Figure 5:**
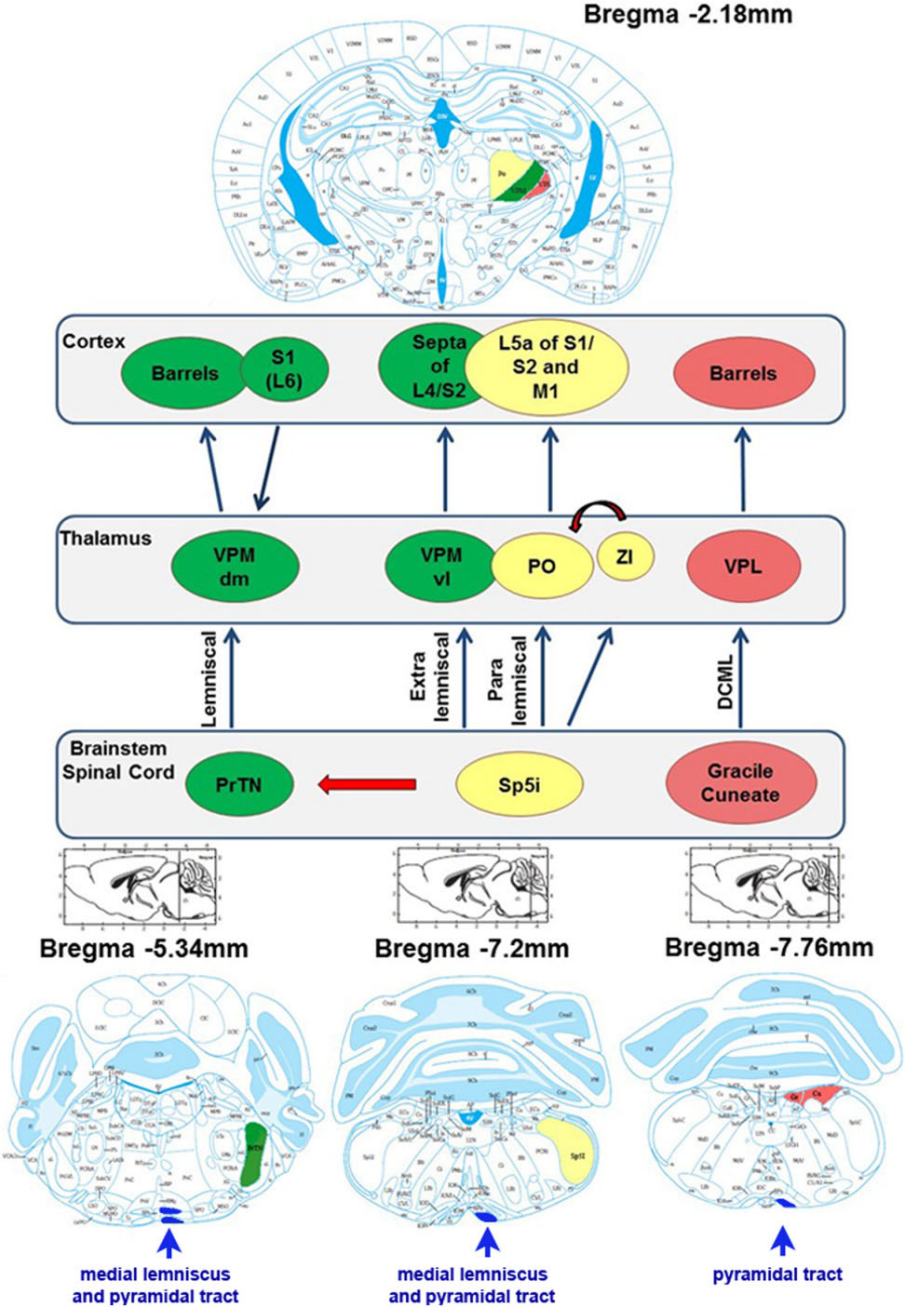
Simplified schematic of the somatosensory thalamus. In the lemniscal pathway, neurons in the principal trigeminal nucleus project to the dorsomedial section of the thalamic ventral posterior medial nucleus (VPMdm), which in turn projects to the primary somatosensory cortex (S1), where they terminate in ‘barrels’, dense clusters of small neurons in layer IV. In the extralemniscal pathway, neurons in the caudal part of the interpolar spinal trigeminal nucleus (Sp5I) are clustered into whisker‐related barreletes and project to the ventrolateral domain of the VPM (VPMvl). The axons of VPMvl neurons project to the septa between the barrels of S1 and to the secondary somatosensory cortex (S2). In the paralemniscal pathway, neurons in the rostral part of the interpolar spinal trigeminal nucleus project, among other targets, to the medial sector of the posterior nucleus (PO) and to the zona incerta (ZI). The axons of PO neurons project to layer V of S1, S2 and to the primary motor cortex (MC). The ventral posterior lateral (VPL), receives inputs from gracile and cuneate nuclei and projects to ‘barrels’ in the somatosensory cortex. The medial lemniscal and pyramidal tracts are indicated in blue near the ventral surface at each rostrocaudal location. Anatomical schemes (from Paxinos & Watson) show the regions specified on the map of sections in different rostrocaudal positions relative to bregma.

Given the very robust thalamic neurodegeneration and the subregional specificity of neuropathological features within the thalamus, we sought to identify key structures that are neuroanatomically connected to the thalamic posterior, ventral posterolateral and ventral posteromedial nuclei. The vibrissal system of rodents is responsible for environmental exploration and for detection, localization and identification of objects by vibrissae movements (whisking) showing relevant interaction between motor and sensory signs in the brain. The first‐order neurons, which innervate the face, have the cell bodies in the trigeminal ganglion and project to primary central afferents, forming the trigeminal tract, in the brainstem trigeminal sensory nucleus. In the brainstem, collaterals from the primary afferent axons innervate second‐order neurons in the PrTN and the interpolari spinal nucleus (Sp5I). Both nuclei project to the contralateral thalamic VPM through the lemniscal and extralemniscal pathways, and the Sp5I also projects to the PO, via the paralemniscal pathway. Then, the projections from the thalamus arrive in the somatosensory cortex. However, these connections are part of a complex network, ascending through these pathways and then descending back to the whisker through motor pathways [23]. A simplified schematic of this network is illustrated in Figure 5.

**Figure 6:**
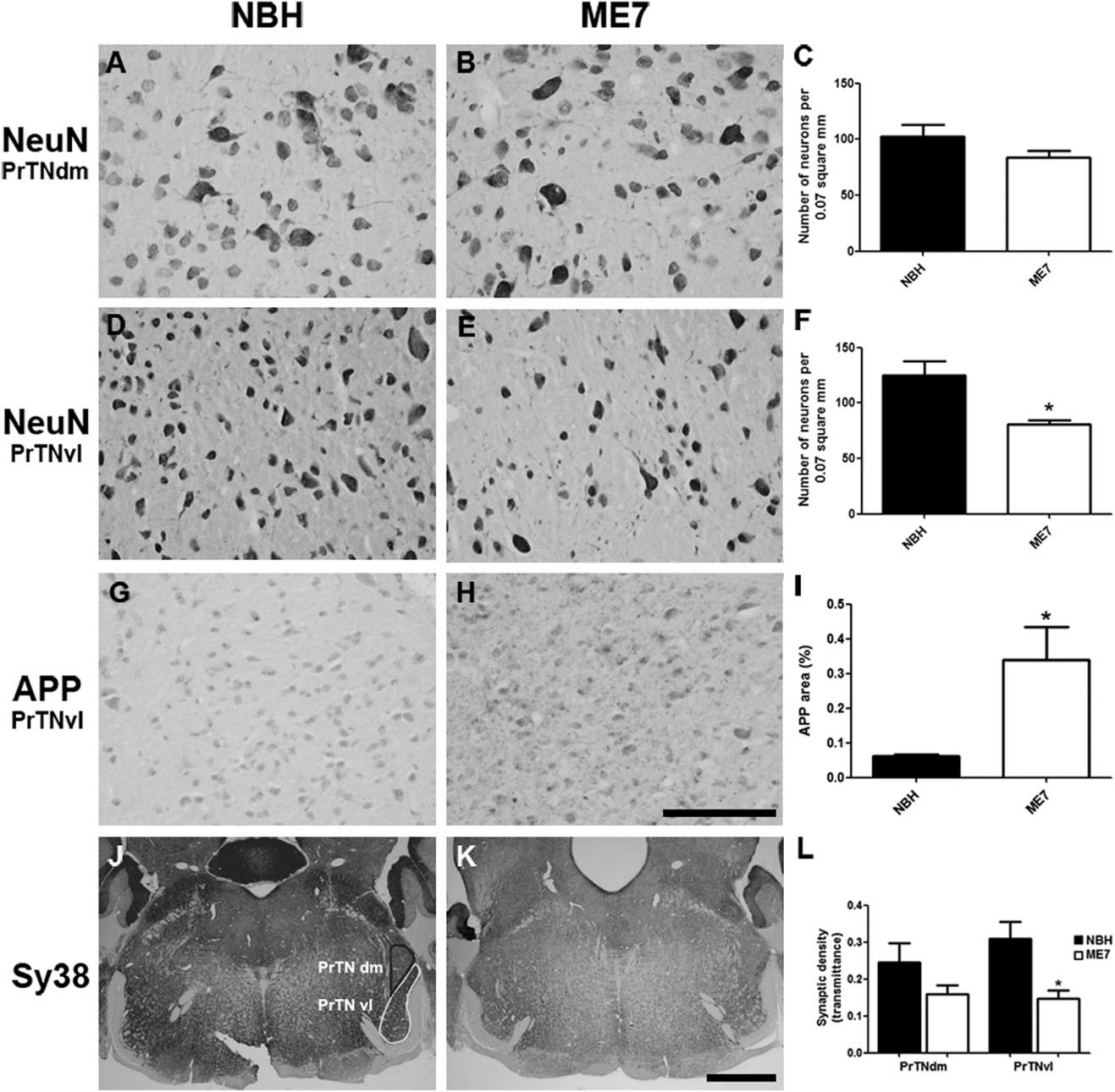
Neuronal, APP and synaptic pathology in the principal trigeminal nucleus. (A–F) Micrographs illustrating NeuN labelling in PrTNdm and PrTNvl of NBH (A,D) and ME7 (B,E) animals, respectively. Masks used to quantify the number of neurons per area, generated in ImageJ software, are shown in supplemental material. ME7 induced significant neuronal loss in PrTNvl (F) but not in PrTNdm (C). *t‐test, *P* < 0.01. *n* = 7 ME7, *n* = 6 NBH. Scale bar: 100 μm. (G–I) Immunolabelling for APP in the PrTN of NBH (G) and ME7 (H). Greyscale images were treated to differentiate brown and blue labelling (see methods) and then thresholded in ImageJ software in order to quantifty APP deposits only. The percentage of APP‐labelled area in the PrTN of NBH and ME7 animals (*n* = 4 in each group) is shown in (I). *t‐test, *P* < 0.05. (J–L) Immunolabelling for Sy38 in PrTN of NBH (J) and ME7 animals (K). The regions of PrTN: ventrolateral part (in white) and dorsomedial part (in black) are delineated in (J). Contrast indices are shown for synaptophysin transmittance measurements in PrTNvl and PrTNdm of NBH (J) and ME7 (K) groups. Statistically significant reduction of synaptic density was found in PrTNvl, but not in PrTNdm (L). *t‐test, *P* < 0.01. PrTNVL, principal trigeminal nucleus, ventrolateral part; PrTNdm, principal trigeminal nucleus, dorsomedial part. *n* = 10 ME7 and 6 NBH. Scale bar: 1 mm. All data are shown as mean ± SEM.

As the PrTN is the principal projection area to VPM, immunohistochemistry for NeuN was performed to see if this region is compromised concurrent with massive synaptic loss in the VPM. Neurons showed different morphology in the dorsomedial (PrTNdm) and ventrolateral (PrTNvl) regions (Figure 6 **A,B**) permitting delineation of the subregions of PrTN for quantification. From micrographs of each region of NBH and ME7 animals the number of neurons per unit area was estimated as shown in the methods (ImageJ masks used for quantification are shown in Supporting Information Figure S1). Micrographs of the dorsomedial area (Figure 6 **A,B**) did not show a significant difference between groups for number of neurons per unit area (Figure 6 **C**). However, the ventrolateral part (Figure 6 **D,E**) showed a statistically significant reduction of neurons in infected animals compared with normal animals (Figure 6 **F**, *P* < 0.01). Quantitative analysis of neuronal labelling was also performed in the Sp5I region, but no difference between the groups was observed (data not shown).

Consistent with the neuronal reduction found in PrTNvl, immunohistochemistry for APP in this region also showed numerous APP deposits in ME7 animals particularly in this region, although small numbers of granules can be found in another regions. In NBH animals, very few granules were found throughout this region. Figure 6 shows the 8‐bit‐converted micrographs from NBH (Figure 6 **G**) and ME7 (6H), and the resulting quantification using ImageJ software. The percentage of the field that was APP positive is shown in Figure 6 **I**, confirming statistically significant difference between the groups (*P < 0.05). Similar APP pathology was not observed in Sp5I nucleus (data not shown), suggesting that this APP pathology occurs in regions where the neuronal cell soma is lost (that is, PO and PrTNvl, but not Sp5I).

As robust differences were found between normal and diseased animals in the PrTN, immunolabelling for synaptophysin was also performed in these tissues. As the PrTN is an important projection area to thalamic VPM, via the lemniscal pathway, density analysis was done on the both sub regions of this nucleus (PrTNvl and PrTNdm; J *vs.* K) and revealed a significant reduction of transmittance only in PrTNvl (6L, *P* < 0.01) although there is a trend towards reduction in PrTNdm. However, micrographs of the brainstem (Bregma −5.2) show that there is a considerable decrease in synaptic labelling in infected animals in most regions at this brainstem level (Figure 6 **J *vs.* K**).

**Figure 7:**
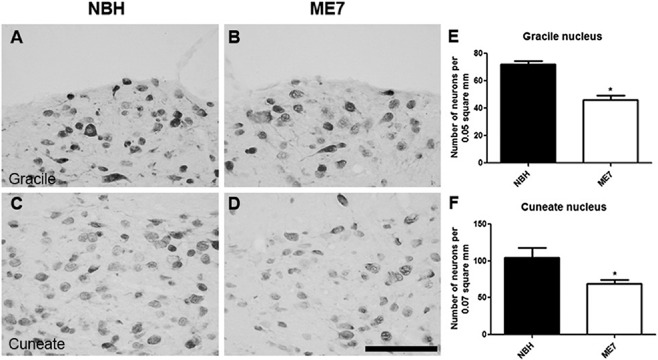
Neurodegeneration in the gracile and cuneate nuclei. Immunolabelling for NeuN in gracile and cuneate nuclei of NBH (A,C) and ME7 (B,D). Both graphs for gracile (E) and cuneate (F) revealed statistically significant reduction of neurons in ME7 animals (*t*‐test, *P < 0.05; **P < 0.01). Data are shown as mean ± SEM with *n* = 4 in each group. Scale bar: 100 μm.

The most important projection to the thalamic VPL originates in the gracile and cuneate nuclei. As the VPL shows dramatic synaptic loss in the ME7 model (Figure 2), we hypothesized that the major population projecting to the VPL would show significant neuropathology. To test this hypothesis, immunohistochemistry for NeuN was performed on gracile and cuneate nuclei of NBH and ME7 animals (Figure 7). Micrographs from normal and infected animals demonstrate a significant reduction of neurons in ME7 animals in the gracile (Figure 7 **B**) and in the cuneate (Figure 7 **D**). The quantitative results confirmed statistically significant neuronal loss in the gracile (Figure 7 **E**) and in the cuneate (Figure 7 **F**) of diseased animals (*P* < 0.05, *t*‐test).

**Figure 8:**
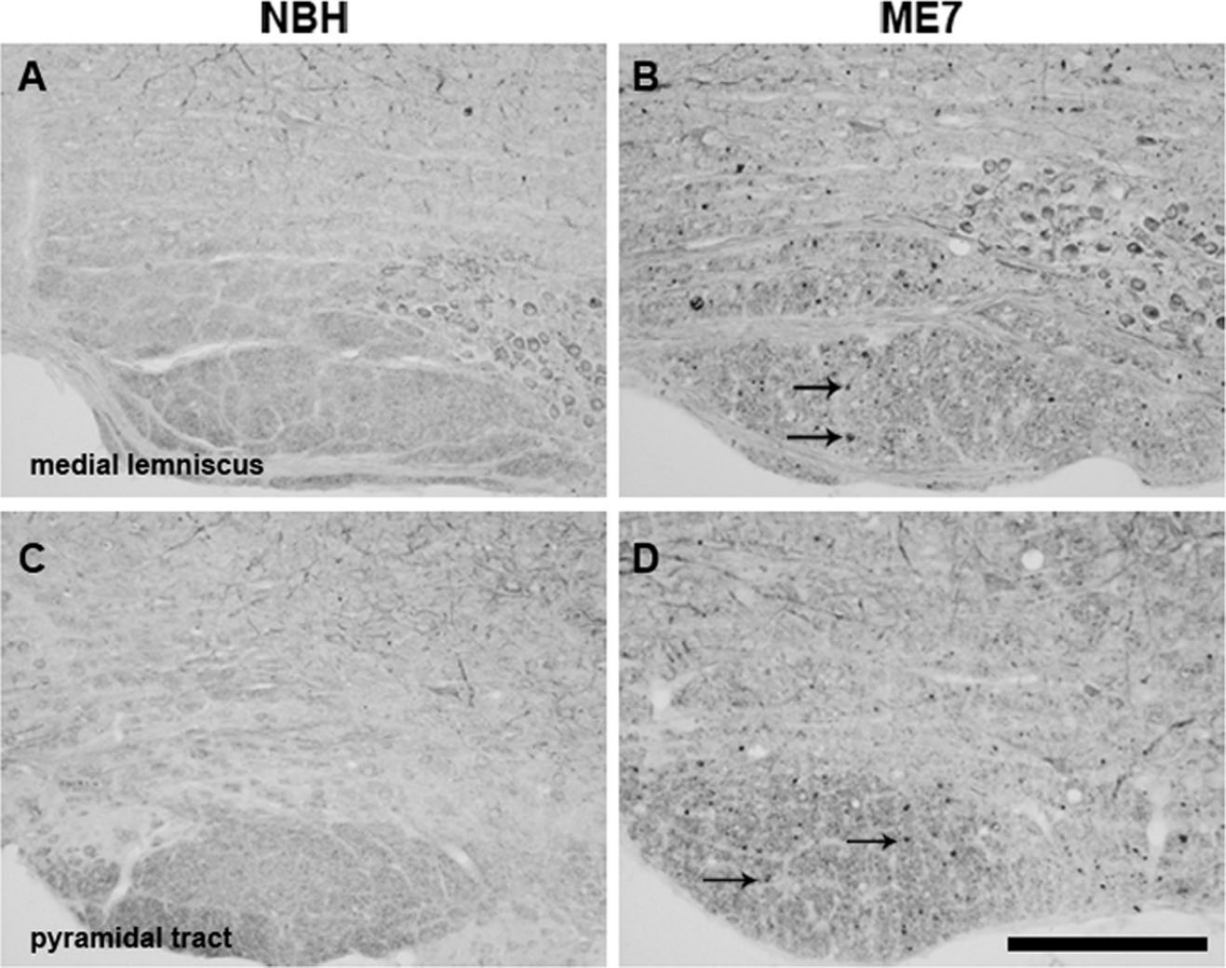
Axonal pathology in the medial lemniscus and pyramidal tract. Immunolabelling for SMI32 in coronal sections, at the rostrocaudal level, containing PrTN and Sp5I of experimental groups. High magnification images of medial lemniscus (B) and the pyramidal tract (D) show significant white matter pathology in ME7 animals, as indicated by intense SMI32 labelling (denoted by black arrows) that is not present in NBH (A,C). PrTN, principal trigeminal nucleus; Sp5I, spinal trigeminal nucleus, part interpolari. Scale bar = 200 μm.

### Axonal injury

To assess for axonal injury in ME7 animals, immunolabelling for nonphosphorylated neurofilament H (SMI32) was performed on PrTN and Sp5I regions in both groups (Figure 8). Nonphosphorylated neurofilament recognized by SMI32 revealed labelling of some neuronal cell bodies and some cell branches but these were similar in both groups. No differences between the groups were found in PrTN and Sp5I nuclei (not shown); however, clear abnormal SMI‐32 labelling was found in the medial lemniscus of ME7 compared with NBH animals (examples shown by arrows on Figure 8 **B**). Although functionally distinct, the lemniscal, extralemniscal and paralemniscal pathways are all carried in the medial lemniscus so it is likely that there is abnormal neurofilament labelling in the axonal tracts from the trigeminal nuclei to all three thalamic areas studied here. Interestingly, abnormal deposits are also found in the pyramidal tract of ME7 animals (arrows on Figure 8 **D**) that suggest a compromise of descending motor pathways originating in the cerebral cortex.

## Discussion

We demonstrate severe thalamic degeneration with marked neuronal loss in the posterior nucleus and widespread degeneration of presynaptic terminals that is most marked in the VPM. Intense deposition of APP was also observed in the PO and VPL. Despite the differential pattern of neuronal pathology across these discrete thalamic nuclei, PrP^Sc^ deposition and microglial activation appeared relatively uniform across these three structures, emphasizing the selective vulnerability of particular neuronal structures despite equivalent levels of PrP^Sc^. We also found significant axonal pathology in the major axonal tract projecting to these thalamic nuclei (medial lemniscus) and significant neuronal loss in some (PrTNvl, gracile and cuneate) but not all (SpI5) of the brainstem nuclei from which these axons originate. Therefore, the thalamus is a major site of neuronal pathology in the ME7 model, allowing us to illustrate discrete neurodegenerative processes in thalamic subregions and in neuroanatomically linked brainstem areas.

The pathology observed here may provide insights for basic processes of neurodegeneration. The most striking novel pathology described here is the very dense APP labelling in the thalamus, particularly in the PO, where neuronal loss was most severe, and in the VPL, with relative sparing of the VPM, where synaptic loss was most severe. This APP pattern is absent in the hippocampus and we find limited evidence of this pathology elsewhere. The APP deposits do not resemble the classical axonal end‐bulbs of axonal transection, suggesting a distinct type of axonal/neuritic stress, and do not resemble PrP^Sc^ labelling in size or distribution. Double‐labelling experiments showed some co‐localization with the axonal marker SMI31, suggesting axonal spheroids, and some of those observed do have a ‘beads on a string’ appearance (Figure 4 **D**). Many of the APP‐positive puncta appeared to be extracellular and may be consistent with the extrusion of axonally localized aggregated proteins or varicosities as an early consequence of neuronal/axonal stress [24]. These extruded varicosities may remain in the extracellular space and may seed further amyloid, while others can be engulfed by local phagocytes, including microglial cells and astrocytes [25]. Consistent with this idea, some APP deposits co‐localized with the lysosomal marker cathepsin D and some with IBA‐1‐positive microglia, suggesting phagocytosis. Although co‐localization with cathepsin D could theoretically occur in any cell type, it is clear that there is a significant shift from predominantly neuronal cathepsin D in the thalamus of NBH animals to largely phagocyte cathepsin D in ME7 animals (Figure 3 **F,G**) and the observed double‐labelled structures were generally larger and localized to glia (Figure 4 **H**).

In the Knuesel and Krstic hypothesis of neurodegeneration [25], axonal APP build‐up is an early event that leads to axonal dysfunction and later tau hyperphosphorylation and tangle formation. Like some earlier studies in rodent and human prion diseases [26, 27], we also observe significant tau hyperphosphorylation and this is predominantly neuritic in the thalamus (Figure 3 **D**) and in the lateral septum and the mossy fibre pathway of the hippocampus (data not shown). However, the most striking axonal SMI32 labelling tended to occur where axons emerge from the medial lemniscus into the posterior nuclei (Figure 3 **E**), suggesting that the grey matter APP labelling could also be non‐axonal. There is evidence that extracellular N‐terminal APP fragments can contribute to neuronal death via the DR6 receptor and caspase 6 and this merits investigation in the current model [28, 29].

Neuritic or dendritic beading and/or fragmentation is another possibility, often observed in neurodegenerative diseases [30] and experimentally upon axonal dysfunction [31]. At least some of the labelling (SMI31/32) observed here (Figure 3 **E**) resembles that seen in experimental autoimmune encephalomyelitis [30] and the observed APP labelling resembles neuritic beading induced by hypoxia/excitotoxicity in cultured neurons [32]. There is significant ubiquitination in the thalamic nuclei in the current study (Figure 3 **C**) and this also has a widely distributed and punctate appearance. Although ubiquitin does not show the same subregion selectivity as the APP pathology, it does resemble the previously described perisomatic ubiquitin granules of 1–4 μm [33, 34], first described as nonplaque dystrophic neuritis [35] outside AD and Pick's disease hippocampal neurons. These structures are thought to be dendritic and are also distinct from ‘dot‐like’ ubiqutin deposits found predominantly in the white matter [36].

Collectively, APP and SMI32 suggest significant axonal pathology. Despite evidence that axonal function can survive a significant degree of dystrophy during amyloidosis and may still recover normal function [37], it is striking that the APP pathology is most severe in the thalamic PO, which ultimately sustains massive neuronal loss. The region from which the PO receives its major projection (via the paralemniscal pathway), the interpolaris, shows no obvious neuronal loss or APP labelling making it unlikely that PO neuronal pathology is the result of pathology originating in the interpolaris.

The main finding in the VPM was the devastating loss of presynaptic terminals while the region from which it receives its major projection, the PrTN (through the lemniscal pathway), showed significant neuronal and synaptic loss and presence of small APP deposits. The ventrolateral part (PrTNvl), which is composed of smaller glutamatergic neurons than the dorsomedial part (PrTNdm), contains the representation of the whiskers and sinus hairs on the snout [38] and shows significantly greater cell loss. These neurons, likely die by a dying back mechanism, which begins in the presynaptic terminal, as we have already described significant synaptic loss occurring in the VPM beginning between 12 and 15 weeks [39], which precedes the robust neuronal death in these prion models [8, 40].

An initial loss of presynaptic terminals on the dendrites of CA1 neurons in the stratum radiatum of the hippocampus is prominent in the ME7 and other models of prion disease [8, 11, 12, 41] [14]. The CA3 axons lose large numbers of terminals, but it is their target cells, the CA1 pyramidal neurons that die, while CA3 pyramidal neurons are relatively spared. Conversely, the thalamic PO cells die despite relative preservation of presynaptic elements terminating on these cells, while the VPM loses presynaptic terminals in spectacular fashion and the neurons in the brainstem region from which the majority of these presynaptic terminals originate (PrTN) also die (30–40%). Thus, neuronal death in prion disease has different mechanisms depending on the region and neuronal or synaptic type. Previously, robust degeneration of Purkinje cells in the 22L strain was demonstrated and was preceded by destruction of their dendritic arborization and sparing of the presynaptic terminals synapsing on the parallel fibres [12]. At the same time, in the same strain, hippocampal CA1 stratum radiatum presynaptic terminals are lost despite sparing of the post‐synaptic density [12]. Similarly, it has been shown that there is a significant loss of dendritic spines, preceded by dendritic varicosities, in the somatosensory cortex of animals injected intracerebrally with the RML strain [9]. A structural basis for such selectivity of degenerative processes has been proposed [42].

The somatosensory cortex is also the target area for projections from the posterior nuclei of the thalamus and the RML strain shows significant spongiosis in the thalamus [43]. Given the lack of obvious neuronal or glial pathology in the somatosensory cortex in our study, the neuropathological processes occurring there remain of interest. In a cathepsin D^‐/‐^ model of neuronal ceroid‐lipofuscinosis, similar synaptic loss was observed in the VPL/VPM, but pathology was similarly limited in the relevant somatosensory cortex [44]. Given that cathepsin D knockout is sufficient to produce marked VPM/VPL synaptic loss, the role of impaired neuronal lysosomal function in synaptic degeneration is of considerable interest. In the current study, NBH animals showed large numbers of small discrete lysosomes (Figure 3 **F**) while in disease, we observed significant loss of neuronal cathepsin D and a significant increase in glial cathepsin D (Figure 3 **G**), illustrating apparently enlarged and possibly defective lysosomes in the diseased state.

We have not specifically examined the functional consequences of thalamic and brainstem degeneration, although we do present objective measures of late‐stage motor incoordination and loss of muscle strength. The thalamus is primarily thought of as a relay area underpinning sensory function and the trigeminal sensory nuclei are the first processing stage in the vibrissal (whisker) system of rodents. This makes generalizations to higher mammalian species difficult. However, we also found a significant loss of neurons and axonal pathology in the dorsal column nuclei (gracile and cuneate), the major areas projecting to VPL. This may impair axonal transport [45] and communication between brainstem and somatosensory cortices, perhaps affecting cutaneous sensations of touch, pressure, flutter and vibration reaching the primary somatosensory cortex. There are reports of paraesthesia (tingling or numbness) and dysaesthesia (abnormal sensations in the absence of contact) in some human forms of the disease [46]. However, not all forms of prion disease feature somatosensory deficits or thalamic/somatosensory pathology.

There is also evidence for important roles of the PO in attention and arousal: brainstem cholinergic and noradrenergic (locus ceruleus) neurons enervate VPM neurons influencing spontaneous tonic or burst firing of these neurons with effects for neocortical activation states [47]. This thalamic pathology may contribute to cognitive dysfunction and, therefore, neuropathology in the locus ceruleus merits investigation.

The thalamic pathology described here extends our knowledge on neuronal death previously described in this structure in three discrete murine prion disease models [13], while the specific features of brainstem pathology described here are novel. Recently, a comprehensive survey of brainstem pathology in murine prion disease showed early and significant PrP deposition, gliosis and spongiform change in the locus ceruleus, nuclei of the solitary tract and pre‐Botzinger complex [48]. This might suggest that these regions may be among the clinical target areas in prion disease (that is, critical vulnerable areas in which prion‐induced neurodegeneration must occur for clinical disease to develop [49]). However, only associations with descriptive neurological features have been made at this point and neurodegeneration in those nuclei was not assessed in those PrP overexpressing mice inoculated with ME7 or RML in the right parietal lobe [48]. Nonetheless, the brainstem is an obvious target underpinning key autonomic functions and is consistent with studies showing shorter brain incubation periods when inoculating peripherally [50] or spinally [49]. There is also evidence for brainstem pathology in sporadic CJD, with prominent PrP deposition and neuronal loss in the inferior olivary nucleus and in the pontine nuclei, and presence of dystrophic axon terminals in the gracile nucleus [51, 52].

Our data are consistent with the spread of prion‐associated pathology along neuroanatomical pathways, previously shown in the optic tract [6] and the limbic system [8]. Those studies typically map the spread of PrP deposition or gliosis, but here we demonstrated spread of neuronal pathology. It is not clear how pathology reaches the posterior thalamic nuclei and why these neurons are so susceptible, but thereafter, significant pathology in the major brainstem projections to the PO occurs. Not all brainstem regions examined showed neuronal loss, so it is clearly not the case that all brainstem regions are experiencing neuronal loss at this time. Conversely, synaptic loss appeared to be very widespread across the brainstem regions examined here (Figure 6) and it is clear that as disease advances, the structures vulnerable to this degeneration become numerous. Given the limited thalamic pathology observed when inoculation occurs peripherally [50], the thalamus' importance in spread of pathology is likely greater upon intracerebral inoculation. It is intuitive that significant pathology reaching the brainstem may produce the late or ‘clinical’ stage signs of disease, including ataxia, lack of balance, loss of motor coordination and lordosis [16] but, if we are to identify key ‘clinical target areas’, objectively measured late‐stage signs and quantification of neuronal pathology in areas underpinning those specific functions are necessary in order to elucidate the autonomic dysfunction, akinetic mutism and bradykinesia that herald death in humans with prion diseases.

The data also have a relevance to neurodegeneration in more general terms. Synaptic and axonal degeneration are early features of multiple neurodegenerative diseases. The current data demonstrate that different neuronal populations are degenerating by discrete mechanisms in close proximity to each other, despite apparently similar aggregated protein deposition. Elucidating these mechanisms may be beneficial for understanding neurodegeneration in multiple disease states.

## Conflict of interest

The authors declare no conflicts of interest.

## Supporting information


**Figure S1.** Neuronal death in the principal trigeminal nucleus. Micrographs illustrating NeuN staining in PrTNdm and PrTNvl of NBH (A, C) and ME7 (B, D) animals, respectively. Masks used to quantify the number of neurons per area and the percentage of area occupied by neurons, generated in ImageJ software, are also shown. ME7 induced significant neuronal loss in PrTNvl (G,H) but not in PrTNdm (E,F). *t‐test, *P* < 0.01. *n* = 7 ME7, *n* = 6 NBH. Scale bar: 100 μm.

**Figure S2.** APP pathology in principal trigeminal nucleus. Immunolabelling for APP in the PrTN of NBH (A) and ME7 (B) mice counterstained with haematoxylin. Greyscale images were treated (C, D) to differentiate brown and blue labelling (see *Methods*) and then thresholded in ImageJ software in order to quantifty APP deposits only. (G) Quantitative analysis of APP in the principal trigeminal nucleus of NBH and ME7 animals showing the percentage of stained area with APP antibody. Scale bar: 100 μm. *n* = 4 in each group. *t‐test, *P* < 0.05.
