# Supplementary figures and images for: At the centre of neuronal, synaptic and axonal pathology in murine prion disease: degeneration of neuroanatomically linked thalamic and brainstem nuclei
Source: Neuropathol Appl Neurobiol. 2015 May 30;41(6):780–97. doi: 10.1111/nan.12232 (PMC4744702; doi:10.1111/nan.12232)

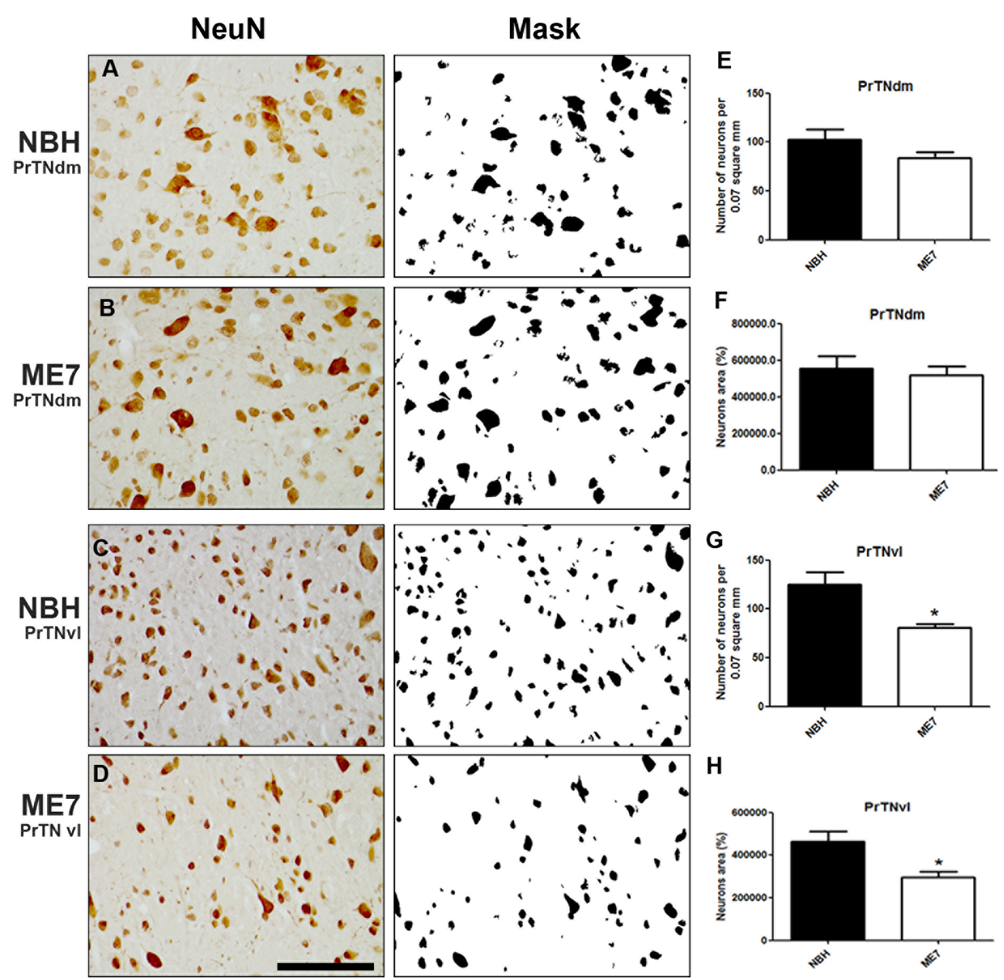

Supplement: Supplementary file 1 — Figure S1. Neuronal death in the principal trigeminal nucleus. Micrographs illustrating NeuN staining in PrTNdm and PrTNvl of NBH (A, C) and ME7 (B, D) animals, respectively. Masks used to quantify the number of neurons per area and the percentage of area occupied by neurons, generated in ImageJ software, are also shown. ME7 induced significant neuronal loss in PrTNvl (G,H) but not in PrTNdm (E,F). *t‐test, P < 0.01. n = 7 ME7, n = 6 NBH. Scale bar: 100 μm. [file NAN-41-780-s001.tif]

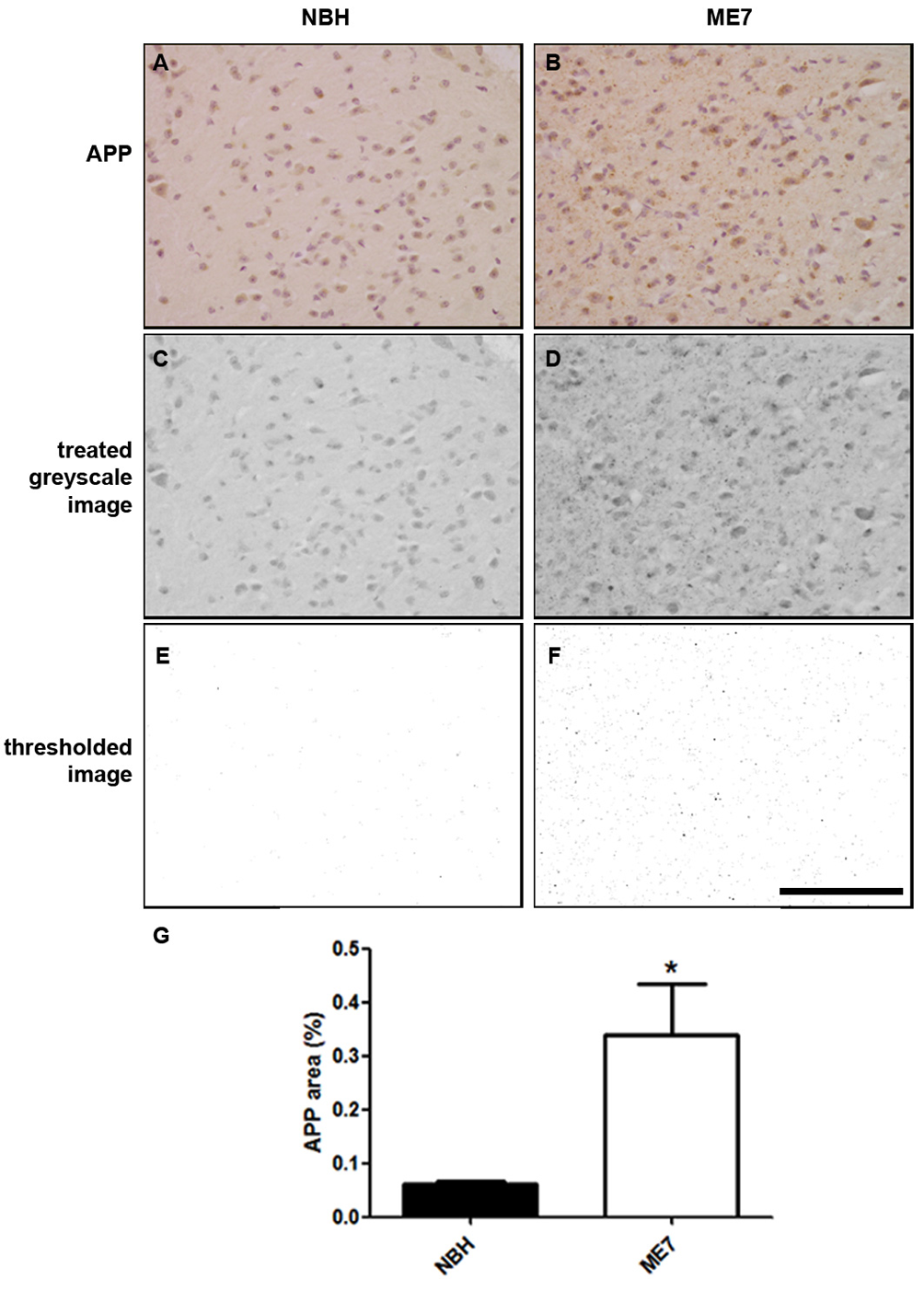

Supplement: Supplementary file 2 — Figure S2. APP pathology in principal trigeminal nucleus. Immunolabelling for APP in the PrTN of NBH (A) and ME7 (B) mice counterstained with haematoxylin. Greyscale images were treated (C, D) to differentiate brown and blue labelling (see Methods) and then thresholded in ImageJ software in order to quantifty APP deposits only. (G) Quantitative analysis of APP in the principal trigeminal nucleus of NBH and ME7 animals showing the percentage of stained area with APP antibody. Scale bar: 100 μm. n = 4 in each group. *t‐test, P < 0.05. [file NAN-41-780-s002.tif]
